# The Resignation of Dr. Bell

**Published:** 1893-10

**Authors:** James B. Bell

**Affiliations:** Boston


### THE RESIGNATION OF DR. BELL.

Boston, September 1st, 1893.

Editor of The Homœopathic Physician :—I notice that my friend, Dr. J. B. S. King, announces me as having resigned from the International Hahnemannian Association, and I would be obliged to you if you would correct the report, as there is no foundation for it.

Yours sincerely,

James B. Bell.

